# Problem Gambling and Psychiatric Comorbidity—Risk and Temporal Sequencing Among Women and Men: Results from the Swelogs Case–Control Study

**DOI:** 10.1007/s10899-019-09851-2

**Published:** 2019-04-25

**Authors:** Kristina Sundqvist, Ingvar Rosendahl

**Affiliations:** 1 Department of Public Health Sciences, Stockholm University, 106 91, Stockholm, Sweden; 2 Department of Clinical Neuroscience, Centre for Psychiatric Research, Karolinska Institutet, and Stockholm Health Care Services, Stockholm County Council, Norra Stationsgatan 69, 113 64 Stockholm, Sweden

**Keywords:** Problem gambling, Comorbidity, Psychiatric conditions, Gambling problems, Gambling disorder

## Abstract

It is well known that many problem gamblers also suffer from other psychiatric conditions. However, knowledge regarding the temporal sequencing of the conditions is lacking, as well as insight in possible gender specific patterns. The aim of this study was to examine the risk for psychiatric comorbidity among problem gamblers compared to non-problem gamblers in the general Swedish population, as well as the age of onset and the temporal sequencing of problem gambling and the comorbid psychiatric conditions among lifetime problem gamblers. A case–control study nested in the Swelogs cohort was used. For both the female and the male problem gamblers, the risk for having had a lifetime psychiatric condition was double or more than double compared to the controls. Having experienced anxiety or depression before gambling onset, constituted a risk for developing problem gambling for the women but not for the men. Further, the female cases initiated gambling after their first period of anxiety, depression and problems with substances, and problem gambling was the last condition to evolve. Opposite this, the male cases initiated gambling before any condition evolved, and depression and suicidal events emerged after problem gambling onset. There were large differences in mean age of onset between the female cases and their controls, this was not the case for the males. Gender specific patterns in the association between problem gambling and psychiatric comorbidity, as well as in the development of problem gambling needs to be considered in treatment planning as well as by the industry in their advertising.

## Introduction

Gambling can be both a fun activity and a destructive and devastating behavior. Gambling engagement is often thought of to be on a continuum, ranging from non-gambling and recreational gambling on one end, to a psychiatric condition—gambling disorder, on the other (Volberg et al. 2015). The term problem gambling is often used to describe gamblers who suffer significant consequences from their gambling, and includes gambling disorder (Neal et al. 2005): “Problem gambling is characterised by difficulties in limiting money and/or time spent on gambling which leads to adverse consequences for the gambler, others, or for the community*”* (p. 3). The prevalence of past-year problem gambling vary between 0.12 and 5.8% across different countries in the world, and the majority (about 75%) of problem gamblers are male (Calado and Griffiths 2016). In Sweden, 1.3% of the adult population are categorized problem gamblers, and of those 0.6% are considered disordered gamblers (Public Health Agency of Sweden, 2019).

The co-occurrence of psychiatric comorbidity is high among problem gamblers. Substance use disorders, but also mood disorders and personality disorders has been linked to problem gambling (Raylu and Oei 2002; Shaffer and Martin 2011). A meta-analysis conclude that the highest mean prevalence of other psychiatric disorders among problem gamblers was for (except for nicotine dependence); substance use disorder (57.5%), mood disorders (37.9%) and anxiety disorders (37.4%) (Lorains et al. 2011). When comparing problem gamblers with non-gamblers, the risk of having a diagnosis of substance use disorder in their lifetimes has been found to be between 5 to 6 times higher for problem gamblers, compared to non-gamblers (Kessler et al. 2008; Petry et al. 2005). Further, the prevalence of mood disorders and anxiety disorders were more than three times higher in problem gamblers compared to non-gamblers.

The association between problem gambling and psychiatric comorbid conditions differ across gender, and has been found to be stronger among women than among men (Desai and Potenza 2008; Petry et al. 2005). A recent register based study on treatment seeking problem gamblers in Sweden found that anxiety and affective disorders were more common among women than among men, whereas there were no significant gender difference regarding alcohol -and substance use (cannabis excluded which was more common among male problem gamblers) (Håkansson et al. 2018). A Finnish population-based study found psychological distress to be associated with an increased risk of problem gambling in men, whereas alcohol-related problems were significantly associated with problem gambling among female gamblers (Nordmyr et al. 2014). In community based studies, mood- or anxiety disorders has been found to predict future problem gambling for women but not for men (Blanco et al. 2006; Desai and Potenza 2008). Connected to this, women more often report gambling to relieve negative emotions (Desai and Potenza 2008; Ibáñez et al. 2003).

Even though it is generally accepted that many psychiatric disorders co-occur with problem gambling (El-Guebaly et al. 2006), conclusions drawn about psychiatric comorbidity in problem and pathological gambling often rely on studies on treatment-seeking samples. However, most problem gamblers never seek professional treatment (Braun et al. 2014), and in addition, treatment-seeking problem gamblers may differ systematically from problem gamblers in the general population. Studies suggests that treatment-seeking samples mainly comprise of white, middle-aged gamblers (Volberg 1994), with more severe problem gambling symptoms and are more likely to have experienced comorbid conditions (Lorains et al. 2011). Given this, results from studies based on treatment-seeking gamblers are less useful for drawing conclusions about problem gambling and psychiatric comorbidity in the general population. Further, in most studies on problem gambling and psychiatric comorbidity, women and men are merged in the analyses, preventing gender specific patterns to show.

Although the reciprocal nature of the association between problem gambling and psychiatric disorders is unclear, it has been indicated that in problem gamblers with a comorbid disorder, the onset of problem gambling preceded the comorbid disorder 23.5% of the time, whereas problem gambling followed the comorbid disorder 74.3% of the time (Kessler et al. 2008). It has also been suggested that mood and anxiety disorders predict the subsequent onset of problem gambling, whereas problem gambling more often seem to predict the subsequent onset of substance use disorders than vice versa.

Recently, Hartmann and Blaszczynski (2018) conducted a review over longitudinal studies on comorbidity between problem gambling and other psychiatric conditions, and found the association to be bidirectional. They also found that impulsivity was a strong predictor of later problem gambling and an underlying interactive factor that can drive both the development of problem gambling and depression. Few of the studies included in the review examined gender differences, but one study included (Barnes et al. 2002), found that alcohol predicted problem gambling for men, but for women only if impulsivity also was present. Further, results from a study on treatment seeking adults revealed that women tended to experience other disorders before the first onset of problem gambling, whereas men tended to experience other disorders after the first onset of problem gambling (Haw and Holdsworth 2016).

The issue of temporal sequencing is not only of interest for treatment planning, but also for the development of harm minimization strategies. Within the gambling industry, some holds the opinion that the product itself is fairly safe, and that people developing gambling problems do so due to underlying psychiatric vulnerability, whereas others argue that gambling can lead to psychiatric issues, and needs to be considered when debating the impact of gambling on society (Delfabbro and King 2012).

The aim of this study is to examine the risk for psychiatric comorbidity among problem gambling women and men, compared to non-problem gamblers, in the general Swedish population. A second aim is to examine the age of onset and the temporal sequencing of problem gambling and the comorbid psychiatric conditions, among lifetime problem gamblers.

## Methods

### Design

The Swedish longitudinal gambling study (Swelogs), is a longitudinal research program on gambling and problem gambling in Sweden (Romild et al. 2014; Statens Folkhälsoinstitut 2013). It was initiated in 2008, and is managed by the Public Health Agency of Sweden. The program has two tracks, the Epidemiological track (EP), in which four waves of data collection have been carried out and the In-Depth (ID) track, in which three waves of data collection have been conducted. The main purpose of the EP track is to estimate the prevalence and incidence of problem gambling in the Swedish population.

The ID track is a case–control study within the Swelogs cohort, with the purpose of collecting information about the mental health of the study participants in a lifetime perspective. The cases were defined as participants in the Swelogs cohort who had ever experienced problem gambling (scoring 3 or more on PGSI 12 months or on SOGS-R life, n = 591), and agreeing to participate in the ID1. The controls (n = 2400) were frequency-matched to the cases based on sex and age, with approximately three controls for each case. Two data collections have been carried out (2011 and 2013). The planned third wave was transformed into a qualitative study (Samuelsson et al. 2018) and therefore not included in the present study. Data was collected through telephone interviews among cases and controls, conducted by the Centre for Psychiatry Research at Karolinska Institutet, and through postal questionnaires among non-participants in the interview. The interviews covered gambling related issues, a psychiatric diagnostic assessment, life stressors and adverse events, family and participant socio-demographic aspects. Socio-demographic information from official registers was linked to the data set.

All participants were sent information per mail about the study. They were informed that participation was voluntarily and what register should be linked to the collected interview data. Respondent were offered 200 SEK (about 22.5 USD) to participate. All interviewers were educated and had clinical experience, and followed an internet based structured interview guide. Multiple contact attempts (mean = 5.5, max = 25) was made before the respondent was declared not reachable via phone. Respondents who could not be reached via phone were sent a postal survey, with two reminders. The interview guide was pilot tested, and the interviewers got supervision continuously to ensure similar interpretations of answers. Kappa was between 0.48 and 1.00, indicating a moderate to perfect interrater reliability. In addition, 10% of the respondents were redialed to determine that the interview actually took place and was conducted in a satisfactory way. In total 97.7% of the respondents were completely satisfied with the interview.

### Measures

#### Gambling

Swelogs covers numerous questions on gambling, including The South Oaks Gambling Screen-Revised Life Time measure (SOGS-R Life) and the Problem Gambling Severity Index (PGSI; Ferris and Wynne 2001). The SOGS instrument was originally developed to diagnose pathological gambling in clinical settings among adults (Lesieur and Blume 1987). In Swelogs, the SOGS-R was included to enable comparisons with the large amount of previous studies that have used the instrument, and because it includes problem gambling occurring at any time in life, which other instruments often do not. The psychometric properties of the instrument have been evaluated with satisfactory results (Lesieur and Blume 1987; Stinchfield 2002). To measure problem gambling in the past 12 months the PGSI was used. The PGSI was administered to respondents who had reported any gambling in the past 12 months. The PGSI was developed to measure problem gambling among adults in the general population, and aims to measure problem gambling from a public health perspective, focusing on harm and consequences, including the social and environmental aspects of gambling problems (Ferris and Wynne 2001). The psychometric properties of the PGSI find a high internal reliability (Holtgraves 2009; Orford et al. 2010). It is recommended that, based on the sum-score, respondents are categorized into: non-problem gambling (0), low-risk gambling (1–2), moderate risk gambling (3–7), and problem gambling (8+) (Ferris and Wynne 2001). In practice, the categories problem gambling and moderate risk gambling are often collapsed to one, to increase statistical power. In this study, the categories with a score of 3–7 and 8–27 was collapsed to one category, Problem gambling, because there were too few study participants with a score of 8–27.

Based on information about when the participants’ first wagered money of their own, from the first interview of the Swelogs cohort, age at gambling onset was defined. For those who did not remember, the information was considered missing.

#### Psychiatric Comorbidity

To study the prevalence of mood- anxiety and substance use disorders, the subscales on depression, panic syndrome, social phobia, post-traumatic stress disorder, generalized anxiety disorder, alcohol use disorder and substance use disorders from the diagnostic instrument Mini International Neuropsychiatric Interview (MINI) (Sheehan 1994; Allgulander et al. 2009) was used. MINI has been validated in several countries (Lecrubier et al. 1997). Questions are answered with yes/no, and interviewers follow a manual for assessment. Most questions in MINI concern current issues, therefore lifetime questions were added to be able to assess lifetime problems. Also, 3 questions about suicidal events was added. Generalized anxiety disorder, panic disorder, social phobia and post-traumatic stress disorder were combined into ‘anxiety disorders’. The information about diagnoses were combined into dichotomous variables (never/ever). To be able to determine age of onset, for every condition, questions in line with ‘How old were you when you had your first period of depression’ were asked. In the second wave of ID (ID2), the same question was asked regarding gambling; ‘How old were you when you first experienced consequences from your gambling’.

The full interview guide is published in Swedish in the report based on the first wave (Statens Folkhälsoinstitut 2013). Minor changes were necessary to undertake in the postal survey. Among other, MINI had to be replaced with other measures. For that reason, answers from the postal survey are not included in this study.

#### Response Rate and Attrition

During fall 2011, 1876 interviews were conducted, giving a response rate of 78.2%. A larger proportion of the controls responded, compared to the cases (87.5% vs 72.3%). There were no differences in response rate across gender.

The analyze sample for the age of onset (AOO)-analyses consisted of those participating in both ID 1 and ID 2 (N = 1588), since the questions about age of onset for mental illness were asked in ID 1, whereas the question about how old they were when they first experienced negative consequences from gambling were asked in ID2. Only 25% of the cases (n = 110 of 427) reported an AOO for problem gambling. Significantly more of the cases that had reported an AOO for problem gambling had experienced depression (40% compared to 26.1%), Alcohol Use Disorder (AUD; 45.8% compared to 30.9%), Substance Use Disorder (SUD; 16.3% compared to 5.6%), suicidal events (31.8% compared to 21.7%), anxiety disorders (29.8% compared to 19.3%), and any psychiatric disorder (61.8% compared to 46.9%). The groups also differed significantly regarding mean PGSI scores in ID1 (3.35 compared to 1.44) and in ID2 (1.93 compared to .54). Finally, the gender distribution differed significantly between the two groups. Within the group that reported AOO for gambling was 20% females, whereas in the group that did not report an AOO was 38.5 females. No significant difference was found in mean AOO for the conditions between the cases with/without reported AOO for problem gambling. Neither was there any significant difference in proportion with higher education. In short, more of the participants with a reported AOO for problem gambling were men, were worse off regarding psychiatric comorbidity and had experienced more severe gambling problems.

## Analyses

As common in case–control studies, associations (in this case between problem gambling and other psychiatric conditions) were estimated using odds ratios. Since case–control studies are typically done when the study outcome is uncommon in the population, odds ratios will approximate risk ratios (Kelsey et al. 1996; MacMahon and Pugh 1970). Since the matching between cases and controls was based on gender and age, those factors were kept constant.

To outline the sequence of onset of the conditions, a calculation of mean AOO for each condition was conducted. In a next step, to examine the temporal priority in AOO, proportion of cases reporting the problem gambling to be initiated before, after, or at the same year as the other psychiatric condition was estimated.

## Results

The first part of this section describes the risk for psychiatric comorbidity among problem gambling women and men compared to their controls. For part one, respondents from ID 1 was used, consisting of 427 cases (34% females) and 1583 controls (35% females). Of the controls, 37.8% reported having had any lifetime psychiatric condition, the corresponding number for the cases was 60.6% (χ^2^ = 64.8, *df* = 1, *p* < 0.001).

The second part describes the AOO for each condition and the temporal sequencing of the conditions. For the second part respondents participating in both ID 1 and ID 2, whom were asked the MINI, was used (N = 272 cases and 1101 controls).

### Problem Gambling and Psychiatric Comorbidity

#### Depression and Suicidality

The risk for having had a depression, both lifetime and before gambling onset, was twice as big (OR = 2.0) for the cases compared to the controls (see Table 1). It was more common among women to have experienced a depression. More of the female cases (18.0%) had experienced depression before the gambling onset, compared to the male cases (3.0%). The association between depression before gambling onset and problem gambling was statistically significant for women, but not for men.

**Table 1:** Proportion of cases and controls with assessed *lifetime* psychiatric conditions (and *before gambling onset*), odds ratios and 95% confidence intervals

	Case (%)	Control (%)	OR	95% CI
*Depr.*				
Females	48.9 (18.0)	30.4 (7.8)	2.2 (2.6)	1.5–3.3 (1.3–4.9)
Males	25.4 (3.0)	14.8 (2.5)	2.0 (1.2)	1.4–2.8 (0.4–3.0)
Total	33.3 (7.8)	20.2 (4.2)	2.0 (1.9)	1.5–2.5 (1.2–3.2)
*Sucid.*				
Females	42.1 (13.5)	21.2 (6.9)	2.7 (2.1)	1.8–4.1 (1.1–4.0)
Males	17.2 (2.7)	9.4 (1.7)	2.0 (1.6)	1.3–3.0 (0.6–4.2)
Total	25.6 (6.4)	13.4 (3.5)	2.2 (1.9)	1.7–2.9 (1.1–3.1)
*Anx.*				
Females	38.8 (17.7)	22.2 (6.5)	2.2 (3.1)	1.4–3.4 (1.6–5.8)
Males	17.2 (4.3)	10.7 (3.9)	1.7 (1.1)	1.2–2.6 (0.5–2.3)
Total	24.6 (8.7)	14.7 (4.7)	1.9 (1.9)	1.4–2.5 (1.2–3.0)
*AUD*				
Females	26.1 (3.7)	10.5 (1.5)	3.0 (2.6)	1.8–5.0 (0.5–10.4)
Males	38.5 (5.9)	18.7 (3.3)	2.7 (1.9)	2.0–3.7 (0.8–3.9)
Total	34.3 (5.1)	15.9 (2.6)	2.8 (2.0)	2.1–3.6 (1.0–3.8)
*SUD*				
Females	8.2 (1.6)	3.8 (0.6)	2.3 (2.6)	0.9–5.2 (0.2–23.2)
Males	7.4 (0.4)	3.6 (0.7)	2.1 (0.5)	1.1–3.9 (0.0–4.2)
Total	7.7 (0.8)	3.7 (0.7)	2.2 (1.1)	1.3–3.5 (0.2–4.4)

Stratified on gender. N = 427 cases and 1583 controls

Suicidal events were a little more than twice as common (OR = 2.2) among cases than among controls. For both cases and controls, suicidality was more common among the women. Of the female cases 13.5% had experienced suicidal events before gambling onset, whereas only 2.7% of the male cases had the same experience. This association was statistically significant for the women, but not for the men.

#### Anxiety

The anxiety syndromes (generalized anxiety disorder, panic disorder, social phobia, and post-traumatic stress disorder) were differently common among the cases (4.1, 7.5, 6.5 and 3.1% respectively for men and 7.0, 21.1, 15.5, and 12.6% for women). They were also differently associated with problem gambling (30–150% larger risk for the male cases compared to the male controls, and 50–180% larger risk for female cases compared to female controls).

In Table 1, the four anxiety conditions were collapsed into ‘anxiety’. The risk for having had experienced any anxiety condition was twice as large for the cases compared to the controls (OR = 1.9). As for depression and suicidality, anxiety was more common among women compared to men. However, the association between problem gambling and anxiety was almost as strong for men as for women. Of the female cases, 17.7% reported having experienced anxiety syndrome before the gambling onset, but only 4.3% of the male cases. For the female cases who had experienced any anxiety syndrome before gambling onset, the risk of later problem gambling was tripled, compared to their controls. For the male cases, anxiety before gambling onset did not constitute a risk for developing later problem gambling.

#### Alcohol and Other Substances

As shown in Table 1, a little over one-third of the cases had had alcohol problems ever in life. The risk for the cases of having had alcohol problems was almost tripled compared to the controls (OR = 2.8). A little more than 5% of the cases had had alcohol problems before gambling onset, giving a 2 times greater risk for the cases compared to the controls (OR = 2.0). The association between problem gambling and both lifetime alcohol problems and alcohol problems before gambling onset was stronger for the women than for the men. However, the analyses on alcohol problems before gambling onset did not yield any statistically significant differences between the cases and the controls, neither in total nor separated on gender.

Regarding other substances, the risk for lifetime problems was twice as big for the cases compared to the controls (OR = 2.2). The association between substance use problems before gambling onset, and later problem gambling was stronger for women than for men, however neither association was statistically significant. Few cases had experienced problems with substances, hence the estimates are unstable.

### Temporal Sequencing and AOO of Problem Gambling and Other Psychiatric Conditions

**Fig. 1:**
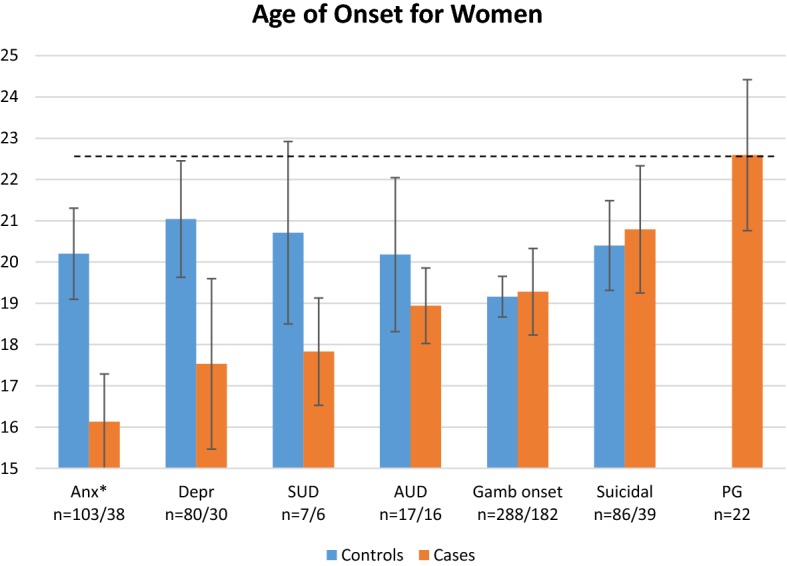
Mean age of onset for *female* cases and controls and standard errors. The dotted line marks the mean age for problem gambling among the female cases

The reported mean AOO for each condition for *female* cases and controls is shown in Fig. 1. The figure shows the chronological order in which the conditions emerge for the female cases. As shown in the figure, for the cases all conditions, except suicidal events, evolved before gambling onset. Further, for the female cases, problem gambling was the last condition to evolve, at an mean age of *M *= 22.6, *SD *= 8.6.

When comparing female cases with their controls, the mean AOO for the first experienced period of the respective psychiatric condition was lower for the cases compared to the controls regarding anxiety (*M *= 16.1, *SD *= 7.1 and *M *= 20.2, *SD *= 11.2 respectively), depression (*M *= 17.5, *SD *= 11.3 and *M *= 21.0, *SD *= 12.6, SUD (*M *= 17.8, *SD *= 3.2 and *M *= 20.7, *SD *= 5.9) and AUD (*M *= 18.9, *SD *= 3.7 and *M *= 20.1, *SD *= 7.7). However, only the mean AOO for anxiety disorders differed significantly between the cases and controls, *t* (104) = 2.5, *p *= .012. The differences between the cases and the controls regarding gambling onset and first experience of suicidal events were small.

**Fig. 2:**
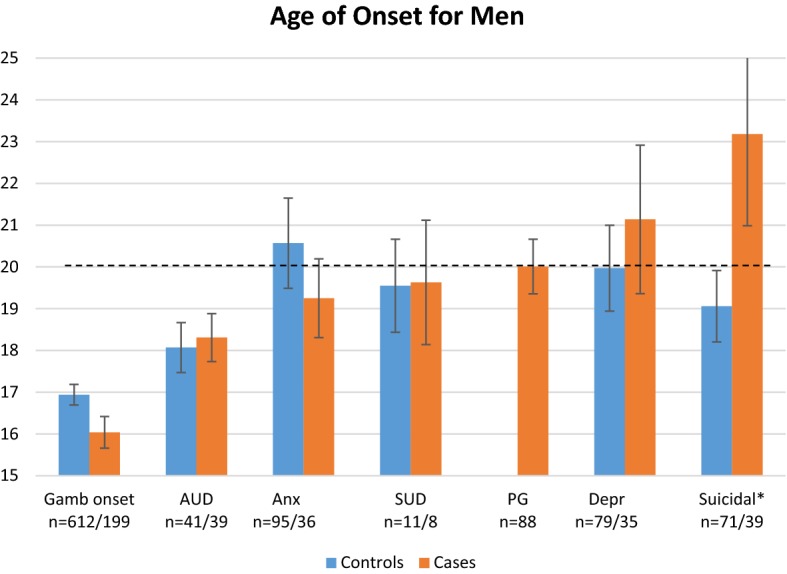
Mean age of onset for *male* cases and controls and standard errors. The dotted line marks the mean age for problem gambling among the male cases

The reported mean AOO for each condition for the *male* cases and controls is shown in Fig. 2. The figures show the chronological order in which the conditions emerge for the male cases. For the male cases, as well as their controls, gambling was initiated before any of the comorbid conditions evolved. The male cases initiated gambling about 1 year earlier than the controls (*M *= 16.0, *SD *= 5.3 and *M *= 16.9, *SD *= 6.1 respectively), experienced periods of anxiety about 1 year *earlier* (*M *= 19.3, *SD *= 5.7 and *M *= 20.6, *SD *= 10.5 respectively), and periods of depression about a year *later* than the controls (*M *= 21.1, *SD *= 10.5 and *M *= 20.0, *SD *= 9.2 respectively), however those differences were not statistically significant. Surprisingly, male cases were significantly *older* than their controls, when they first experienced suicidal events (*M *= 23.2, *SD *= 13.7 and *M *= 19.1, *SD *= 7.2 respectively) *t* (108) = 2.1, *p *= .04.

When comparing women to men, it can be seen that the men initiate their gambling about 3 years earlier than the women. The men also got problems from gambling at an earlier age than the women. There also seem to be a lager difference in mean AOO for the female cases and controls, compared to the male cases and controls.

The scores in the total rows for Table 2 suggest that the majority of participants (58.6–74.3%) who indicated a previous problem with psychiatric conditions reported that the initial onset of problem gambling occurred at an age after problems with the other conditions commenced. For depression, anxiety disorders and suicidal events, more of the women reported the AOO for the psychiatric conditions to be before the AOO for problem gambling. This was the case for 90.9% of the women who had experienced anxiety disorders (and for 66.7% of the men). Among the women, 75% had experience a depression before the onset of problem gambling and among the men 58.6%. The corresponding number for suicidal events were 72.7% for the women and 58.3% for the men.

**Table 2:** Temporal priority in age of onset among cases only

	PG first (%)	95% CI	Other condition first (%)	95% CI	Same age (%)	95% CI
Depression (N = 29) Total	34.5	(19.9–52.7)	58.6	(40.7–74.5)	6.9	(1.9–22.0)
Females 8	25.0	(7.2–59.1)	75.0	(40.9–92.9)	0	(0–32.4)
Males 21	38.1	(20.8–59.1)	52.4	(32.4-.71.7)	9.5	(2.7–28.9)
Suicidal events (N = 35) Total	31.4	(18.6–48.0)	62.9	(46.3–76.8)	5.7	(1.6–18.6)
Females 11	27.3	(9.7–56.6)	72.7	(43.4–90.3)	0.0	(0–25.9)
Males 24	33.3	(18.0–53.3)	58.4	(38.8–75.5)	8.3	(2.3–25.8)
Anxiety disorders (N = 35) Total	17.1	(8.1–32.7)	74.3	(57.9–85.8)	8.6	(3.0-.22.4)
Females 11	0.0	(0–25.9)	90.9	(62.3–98.4)	9.1	(1.62–37.7)
Males 24	25.0	(12.0–44.9)	66.7	(46.7–82.0)	8.3	(2.3–28.5)
Alcohol and substance use disorders (N = 32) Total	28.1	(15.6–45.4)	59.4	(42.3–74.5)	12.5	(5.0–28.1)
Females 7	14.3	(2.6–51.3)	57.1	(25.0–84.2)	28.6	(8.2–64.1)
Males 25	32.0	(17.2–51.6)	60.0	(40.7–76.6)	8.0	(2.2–25.0)

Analyze sample includes individuals participating in both ID1 and ID2, answered MINI and answered age of onset for *both* PG and comorbid disorders

## Discussion

For both men and women, the association between each psychiatric lifetime condition and problem gambling were statistically significant. Compared to the controls, the risk for the cases of having had a lifetime condition was doubled or more than doubled. All conditions were more common among the women, and in addition, the associations between the lifetime conditions and problem gambling were slightly stronger for the women. Alcohol problems were most strongly associated with problem gambling for both women and men, with a 3 times higher risk for the cases compared to the controls. Women who had experienced anxiety or depression before gambling onset had a 2.6–3.1 times higher risk of developing gambling problems later in life, compared to their controls. For men, suffering from any of the conditions before gambling onset did not constitute a statistically significant risk for later problem gambling.

When examining mean AOO for all conditions among the female respondents, gambling onset was later (*M *= 19.3) than the onset of all psychiatric conditions, except for suicidal events. Among the women, problem gambling was the last condition to evolve at a mean age of 22.6. The male respondents started gambling at mean age 16.0, before the onset of any other condition. For the males, problem gambling evolved at a mean age of 20, before both depression and suicidal events.

Further, the majority of the cases who reported previous psychiatric conditions had experienced them before the gambling problems evolved. This was the case for more of the females, than of the males. Even though the mean AOO for depression and suicidal events was higher than mean AOO for problem gambling, among the men, a majority of the men reported that those comorbid conditions evolved before the problem gambling onset.

As in previous studies, the association between problem gambling and comorbid disorders was stronger among women than among men. In this study, the association between anxiety and depression *before* gambling onset and later problem gambling was strong for women, whereas this association was not statistically significant for men. This is in line with previous community-based studies that found mood- or anxiety disorders to predict future problem gambling for women but not for men (Blanco et al. 2006; Desai and Potenza 2008). One possible explanation for this is that not only is anxiety and affective disorders more common among women compared to men in the general population (Piccinelli and Wilkinson 2000; WHO 2001), but there also seen as if the burden of illness are greater for women than for men (McLean et al. 2011). Hence, mental illness constitute a greater risk of developing other disorders among women compared to men.

In the present study, the patterns of AOO were slightly different for the women than for the men, as well as across different conditions. For the women, anxiety preceded problem gambling in almost all cases, and alcohol- and substance use disorders in a little over half of the cases. For the men, anxiety was the most common disorder to precede problem gambling (67%) whereas depression was the least common to precede problem gambling (52%). This is in line with the study by Kessler et al. (2008), where anxiety was the most common disorder to precede problem gambling (82%) and substance use disorders was the least common disorder to precede problem gambling (57%). However, in Kessler et al. (2008) women and men were merged in the analysis, not allowing for additional comparisons. Opposite previous research (Grant et al. 2012; Ibáñez et al. 2003; Ladd and Petry 2002), the female cases in this study did not have a shorter time from gambling onset to problem gambling, compared to the males. Those studies, however, have focused on individuals seeking treatment, which may explain the contradictory results.

In the present study, all comorbid conditions evolved before gambling onset for the women, which can be interpreted as being in line with previous findings that women more often than men gamble to cope with negative emotions (Desai and Potenza 2008; Ibáñez et al. 2003). On the other hand, the women in the present study initiated gambling almost 3 years after the men. The later the gambling onset, the greater the likelihood for having experienced anxiety or depression. Yet again, there seem to be a large difference in AOO for the female cases compared to the controls, a difference that is not present for the males. This suggest, which also is shown in the regression analyses, that women, but not men, with mental illness is a vulnerable group with a large risk of developing problem gambling. The pattern suggests that for women, problem gambling is a consequence of mental illness, whereas for men, problem gambling is part of a process driving a negative evolvement towards depression and suicidal events. Within the field of alcohol research it has been argued that the link between depression and substance use and impairment may be stronger among women than men due to a greater tendency among women to use psychoactive substances as a coping strategy (Conner et al. 2009).

Considering the seemingly greater vulnerability among female gamblers, the responsibility of the gambling companies can be discussed. In Sweden, massive advertising from gambling companies, mainly for different online casinos, has preceded the change from monopoly to a license system (introduced in January 2019). A great deal of those online casinos and campaigns have been designed to attract women specifically. Parallel to this, from 2015 to 2018 there has been an increase in individuals with severe gambling problems in Sweden, and the largest increase has been among women (the Public Health Agency of Sweden, 2019). Even though 75% of problem gamblers in general are male, of the 0.6% of disordered gamblers in Sweden, half are now women.

A major strength of this study is that it uses a large community based sample, making it possible to generalize to the general population. It is also important to elucidate gender specific patterns, since gambling and problem gambling may have different purposes for women and men, and the development of problem gambling may be affected by gender. In addition, the assessment of the respondents mental health by trained interviewers generates more reliable data, as opposed to using self-reported health.

The study suffers from some limitations. Even though the initial sample was large, when using a community sample, the proportion of problem gamblers is usually quite low. Including participants with less severe gambling problems (PGSI +3) is a way to increase statistical power, but at the same time it increases the uncertainty regarding what constitutes a risk gambler (Samuelsson et al. 2018), and whether the behavior of the individuals leads to adverse consequences (Neal et al. 2005). However, few of the cases reported an AOO for problem gambling, and those doing so had more severe gambling problems and higher proportion of comorbid conditions. This indicates that the sample included in the analyses on temporal sequencing was in fact problem gamblers. Further, retrospective reporting of gambling behavior as well as of mental health may be subject to recall bias.

Further research is needed on the casual association between problem gambling and psychiatric comorbidity as well as on gender specific patterns.

## Conclusions

The present study elucidates the different patterns for women and men regarding problem gambling and psychiatric comorbidity. The association seem to be stronger for women, and previous mental illness seem to constitute a risk for later problem gambling for women but not for men. There also seem to be different paths in the temporal sequencing of the psychiatric disorders for women and men. Understanding the different paths for women and men may contribute to the development of treatment that is adapted according to the diverse needs of female and male problem gamblers. It is important that the industry consider the elevated risk of developing problem gambling among vulnerable female (and male) gamblers in their design of advertising.
